# Beyond Brain Drain: Smart Strategies for Government-Led Nurse Exportation in Ghana

**DOI:** 10.1155/jonm/1912530

**Published:** 2025-08-29

**Authors:** Emmanuel Graham Nyameke, John Windie Ansah, Isaac Defiin

**Affiliations:** Department of Sociology and Anthropology, University of Cape Coast, Cape Coast, Ghana

**Keywords:** brain circulation, government-led nurse exportation, institutionalising nurse exportation, nurse export strategies, nurse exportation

## Abstract

The export of nurses or the migration of nurses has predominantly been framed within the discourse of “brain drain,” often overshadowing its potential benefits for nurses, their families and the sending countries. While existing literature highlights the economic and professional gains of nurse migration, few studies explore how structured government-led export strategies could optimise these benefits. This study examines nurses' perspectives on institutionalising nurse exportation through policy frameworks, bilateral agreements, rotational systems, as well as family inclusion. Using a mixed-methods approach, data were collected from 483 nurses in Ghana, revealing strong support (89.4%) for a regulatory framework to govern nurse export. Additionally, 85.7% advocated for bilateral agreements with destination countries, while 82.8% endorsed rotational exportation to mitigate brain drain. A significant majority (83.9%) also supported family inclusion in migration programs. Explaining some issues in the quantitative data, the qualitative data revelled that even though the government could go ahead with the export of nurses guided by a formalised policy, the export of nurses comes with some financial burden as well. The findings further suggest that formalising nurse exports through structured policies could transform migration from an ad-hoc phenomenon into a strategic economic and developmental tool. This study contributes to the discourse on ethical and sustainable nurse exportation, proposing a model that balances global demand with domestic healthcare needs while maximising remittances and skills transfer.

## 1. Introduction

Nurse exportation, a deliberate decision by a government to send nurses abroad from one country to another, is a growing phenomenon in Ghana and other African countries [1]. This service trade has become possible through the growth in regional integration and international diplomatic relations. Also, the overproduction of nurses in Ghana, for example, has necessitated the export of nurses [1]. The global nursing shortage has reached critical levels, with the World Health Organisation (WHO) projecting a deficit of 13 million nurses worldwide by 2030 [2]. This crisis has intensified international competition for skilled healthcare workers, particularly from sub-Saharan Africa where health systems already face severe workforce shortages [3]. Ghana exemplifies this paradox-boasting a robust nursing education system that produces more graduates than its health sector can absorb, while simultaneously struggling with nurse-to-patient ratios that fall far below WHO recommendations [4]. This imbalance has led to increasing interest in structured approaches to nurse migration that could potentially benefit both source and destination countries [5]. The past government of Ghana and the current government have resorted to the export of nurses abroad. Currently, the minister for foreign affairs has signed a bilateral agreement with the Jamaican government to send Ghanaian nurses to work there [6]. While it seems that this move by the government has come to stay, the voices of nurses have not been heard in the area of policy formulation and various strategies that are to be taken, even though their voices remain crucial.

Recent policy developments suggest a shift toward more regulated international nurse mobility frameworks. The UK-Kenya Health Workforce Partnership represents one such model, demonstrating how bilateral agreements can formalise nurse migration while addressing ethical recruitment concerns [7]. Similarly, the WHO Global Code of Practice on the International Recruitment of Health Personnel [2] provides normative guidance for managing health worker migration. However, significant gaps remain in understanding how low- and middle-income countries can develop sustainable nurse export strategies that balance economic opportunities with health system preservation [8].

This study contributes to three critical debates in global health workforce governance. First, it examines the policy infrastructure needed to operationalise government-led nurse export programs, building on Adhikari et al. [9], concept of Global Skill Partnerships. Second, it explores the tension between individual migration rights and collective health system needs—a tension exacerbated by the growing commercialisation of nurse education in many African countries [4]. Finally, it interrogates how rotational migration systems might mitigate brain drain while maximising knowledge transfer.

## 2. Methodology

This study employed a sequential explanatory design, where quantitative data were first collected and analysed, followed by the collection and analysis of qualitative data to further explain the quantitative findings [10]. The rationale for this approach was to provide a general understanding of the research problem through quantitative results before engaging in an in-depth exploration using qualitative interviews [11, 12]. The research was conducted at Cape Coast Nursing and Midwifery College (CCNMC), Psychiatric Nursing Training College-Ankaful (PNTC) and the University of Cape Coast School of Nursing and Midwifery (UCCNMC). These institutions were selected to reflect diverse training backgrounds and to capture a wide range of perspectives on training nurses for potential export.

### 2.1. Population, Sampling and Data Collection

The study targeted final-year students from the three institutions, as they are closest to entering the job market and may be considering migration options [13]. Inclusion criteria required that institutions be government-run, located in Cape Coast, and offer degree or diploma programs in general, psychiatric, midwifery or specialized nursing. Probabilistic sampling, including a lottery technique, was used to select a total of 483 students for the quantitative survey, ensuring diversity and representativeness [14, 15]. For the qualitative component, seven students were interviewed until data saturation was reached, aligning with qualitative research best practices [16]. Data collection instruments included a 63-item questionnaire for the survey and an in-depth interview guide, both developed based on literature review and initial quantitative findings [17, 18]. The interview questions have been attached in the appendix (Appendix, Qualitative instrument).

### 2.2. Data Processing and Analysis

In this study, the researchers used multiple analytical techniques for both the quantitative and qualitative data. The qualitative data were analysed using thematic narrative analysis. The quantitative data were processed, cleaned and analysed using SPSS v.27.

### 2.3. Measures of Frequency

In describing the data in this work, the researchers used frequencies to describe the various variables under themes. Using this type of analysis enabled the researcher to interpret the results using their frequencies and percentages. The measures of frequency analysis enabled the researchers to bring to light the percentages for the various responses. In interpreting the data, the researcher looked at the respective frequencies and percentages. At the end of every interpretation, the researchers presented, percentage-wise, the response for the total sample size for all three institutions to bring to light the responses. The variables were analysed and interpreted under the various themes.

The qualitative data were analysed using thematic narrative and interpretive phenomenological methods. After manual data coding, thematic analysis identified image motifs. Authors found common difficulties among participants by identifying emergent themes [1]. The first researcher listened to the audios and transcribed it by listening, pausing and typing into a word document. The others proofread the transcripts and checked for grammatical errors [1]. The researchers manually coded the transcribed material and produced themes according to Fiaveh [19].

Ethical Considerations: Ethical approval for this research was obtained from the Institutional Review Board of the University of Cape Coast (UCCIRB/CHLS/2023/46) and the Ghana Health Service Ethical Committee (GHS-ERC 048/05/23). Permission was also sought from the heads of participating institutions. All participants gave informed consent, were assured of confidentiality, and were informed of their right to withdraw from the study at any time. Pseudonyms were used in reporting to protect participants' identities, ensuring adherence to ethical research standards [20–22].

## 3. Results and Discussions

The analysis has been presented under four broad themes. After analysing the quantitative data under their specific themes, the qualitative data were analysed under the respective themes. This helped explain the issues in the quantitative data.

### 3.1. Government Nurse Export Strategies

If there is a crop of people who will want to be exported by the government, it is important to know their views on the strategies that the government needs to put in place as far as exporting nurses is concerned. This is what motivated me to ask questions on policy and regulatory framework, arrange with other countries, join TiSA groups, rotate the export and selection process, and include families of nurses in the process.

### 3.2. Policy and Regulatory Framework

From the Table above, the responses suggest that nurses appreciate the need for the government to put in place certain strategies for the export of nurses. These strategies include policy and regulatory framework, arranging with other countries and joining TiSA groups, rotating the export and selection, and including families of nurses in the process. Setting up a policy and a regulatory framework, institutionalising nurse exportation, setting up a regulatory body, and developing a policy to regulate nurse exportation were appreciated with high percentages of 89.4%, 87.4%, and 86.4%, respectively. The high percentages showing agreement communicate that the nurses appreciate the fact that the government would have to have a policy and a regulatory framework to run the export of nurses.

#### 3.2.1. An Avenue for Government to Plan

The interviews also supported the formulation of a policy and regulatory framework. The participants revealed that the government should institutionalise the export of nurses. By institutionalising nurse exportation in Ghana, there should be norms and values to guide the operations. On the issue of whether nurse exportation should be institutionalised or not, it was found that it will become an avenue for the government to plan. Thus, the government will plan on how many nurses need to be trained in order to sustain the local health sector and also the numbers that need to be exported. Further, it was revealed from the interviews that institutionalising nurse exportation will serve as a check and balance for the government. Thus, the government will be able to know the number of health professionals who have been trained, the number who have been sent outside, and make plans on how to stock the health system.

#### 3.2.2. Engage Stakeholder in Policy Formulation

On the issue of formulating policy for the export of nurses, the participants did not only note the importance of policy but they also shared that to come up with the policy, there should be stakeholder engagement. Thus, the various stakeholders should be involved in the draughting of the policy. For the government of Ghana to export nurses, the government may have to have some regional trade policy and some bilateral trade policy with countries that Ghana will trade with. This finding is in line with Maur [23], which revealed that trade policy is important and would have to be put in place between countries that trade among themselves. Further, the benefit of having a policy is that it spells out what the countries want to achieve, how they are going to achieve them, and how they are going to be operationalised. Further, the policy defines what will be done as well as showing how those to be exported will be protected in terms of their rights and work rights. For example, the Kenya-UK and Northern Ireland Agreement (2021) spells out how misunderstandings could be resolved and further explains the motive for the agreement [24].

*The government must look sharp and institutionalise the thing. You see, if the government does it, the office will be making money for the government, and again, they will get to know the number of nurses who have left to help plan the numbers that need to be trained. If the government does not do it, I know nurses will continue to move, so it will be in the best interest of the government to institutionalise it (Mame Abena, a 24-year-old General nurse of UCCSNM).*

*I will be glad if the government will formalise the sending of nurses abroad and take that up. And believe me, I don't think any single nurse will choose to stay behind. It will be a good thing because it will reduce the burden on the government. You know, there are nurses who have not been employed, so if the government should think of sending nurses abroad, it will help the situation. I also believe that it will be helpful because it costs a lot for a nurse to write IELTS exams before the person will get the chance, so if the government is to send nurses, it will mean that all these will be taken care of (Kwamena, a 24-year-old General nurse of UCCSNM).*

*Before anything is done, I believe there must be stakeholder engagement, which will help inform what to do, how to do it, and all the necessary things that need to be considered. Formulation of policy is key to everything. Without policy, everything will be like how things are done in a market. So policy is very important in this, and the government must have a policy to guide the whole export thing (Akosua, a 25-year-old Midwifery nursing student from CCNMC).*

#### 3.2.3. Post All Graduate Nurses Than Institutionalising Nurse Export

A participant had a diverting view on the issue of institutionalising nurse exportation. For her, it is not about exporting nurses, but the government should post all trained nurses. She disclosed that the GHS needs more nurses, and as a result, posting the nurses will help the various health facilities. She, however, noted that the government knows better, so if the government thinks that nurse exportation should be institutionalised, that is okay. This participant demonstrates care for the people of Ghana and would not want them to suffer the consequences of the nurse shortage. This finding can be highlighted by the SI theory that perceiving the consequences that exporting nurses may bring to the local health sector and to the people should not be institutionalised.

*Although I do not agree that the government should establish an institution to send nurses abroad, I cannot stop the government from doing that if the government should think that it will be helpful. What I believe they should do is to train more nurse, post all nurses to the hospitals so that they can help with the work. In fact, Ghana needs more nurses than we think (Memuna, a 27-year-old enroled nurse of CCNMC).*

#### 3.2.4. Export of Nurses Through Bilateral Agreement or Through Agencies

On the issue of arrangement, it was further found from the interviews that the government must communicate with receiving countries to establish business with them to trade in labour. By this, the participants shared that there will be the need to operate the export of nurses within a bilateral agreement. Not only should the export be done among countries, some participants suggested that the government also arrange with other recruiting agencies to send nurses to them. It was found that the MOH can be charged to make the arrangements on behalf of the government. It was revealed from the interviews that arranging with other countries to trade in services promises security. Thus, those to be exported will be assured of their security. The findings agree with literature on bilateral trade agreements that through that module, the labourers are protected and the interests of the partners are safeguarded [25]. Also, the symbolic interactionist theory is at play here since the participants are basing on the benefits of going into a bilateral arrangement, suggesting that the government should go by bilateral agreement if the government is to export nurses.

*I believe it was the government of Kenya and that of the UK who did whatever arrangements to send the nurses to the UK. So, I believe the government of Ghana should be doing every arrangement with countries that will need nurses. The government should be the one to go into contract with other recruiting agencies in the UK, USA, Canada so that the whole process will be clean and fair. Because, when some nurses move on their own, sometimes we hear some of them end up at places less than a hospital (Kwamena, a 24-year-old General nurse of UCCSNM).*

*For me, I think if the export is done by the government that one is better because, with that one, those who will be sent will be safe. So, the government should make all the arrangements. Maybe the Ministry of Health can look out for countries that are in need of nurses and do the negotiation (Akosua, a 25-year-old Midwife of CCNMC).*

### 3.3. Nurse Export on Rotational Basis

On rotational issues, from Table 1, the respondents appreciated the need to rotate the export of nurses. Thus, 82.8%, 79.5% and 77.2% of the respondents agreed that the export of nurses should be done on a rotational basis, and after every 5 years, the nurses should be returned for a new set to be sent. From the interviews, some of the participants did not only reveal their support for the export of nurses, but they unveiled that rotating the export of nurses would help restore the local health facilities with nurses. Thus, it will help check that there are always enough nurses in the various local health sectors to attend to patients.

#### 3.3.1. Knowledge Transfer and Staff Sustainability

The qualitative data further revealed that the rotations of the nurses exportation will lead to knowledge transfer. It was also revealed that when the export is rotated, it will help the hospitals have nurses who have had some experiences from other countries, and that will improve health delivery. Further, rotating the nurses will lead to knowledge transfer. When nurses are rotated within the framework of nurse export, it will enable nurses to gain some foreign experiences with regard to treating diseases and taking care of patients, which will be beneficial to the country. Also, for the participants, rotating the nurses who will be exported will check brain drain. Thus, the country will not outright lose its nurses, but the rotation will be a check balance to always make sure that there are enough nurses to serve in the local health sector. The findings can be understood from the SI theory that the participants perceiving the benefits of rotating nurses when it comes to exporting nurses agreed that rotating the nurses will benefit the local health sector. Hence, they agree that the government can go ahead and export nurses.

*Sending nurses and bringing them back after their services will not be a bad idea. This will help restore the local health sectors with nurses who have experienced how patients are treated in different countries and will transfer the knowledge to their present work. Also, bringing them back will control the rate of nurses lost in the country (Mame Abena, a 24-year-old General nurse of UCCSNM).*

#### 3.3.2. Do Not Rotate the Export of Nurses, Let Them Stay There to Work

From Table 1, on the same issue of rotation, some respondents disagreed with rotating the sending of nurses abroad, and they are being returned after every 5 years (17.2% and 20.5%, respectively). Like we had some respondents disagreeing with the rotations of nurses exported, in the qualitative data, some of the participants also revealed that they do not support the rotation of nurses to be exported. It was found that the nurses who will be sent abroad by the government should be allowed to stay and work until the contract expired. Their reason was that the nurses sent there to work would have become familiar with the work and the environment than sending new set of nurses who would have to now study the hospital setting and the entire environment. Some participants also shared that they will not even want to return when sent abroad to work in another country. This finding could be understood from the perspective of the SI theory in the sense that when individuals perceive that something would not benefit them, they will not want to do it or take action.

*I don't agree that the exportation of nurses should be on rotations. Unless the contracts have expired which will mean that the nurses are to return to the country. Those who will be sent must be allowed to stay and work because they will become familiar with the work there, and so sending another set will mean that they are now going to face a new environment before they get adjusted and begin to work. It will not help (Mame Adwoa, a 24-year-old General nurse of CCNMC).*

### 3.4. Nurse Selection and Family Members Inclusion in the Export Plan

On the selection inclusion of families when it comes to the export of nurses, the data on the Table show that there was a high response in favour of that decision (83.9% for family inclusion and 76.4% for selection being done by the government). Specifically, on the selection of nurses to go, 369 of them out of the 483, representing 76.4%, agreed that the government should do the selection process. To further understand how the selection process should go, the researcher engaged some of the nurses in an interview to explore further.

#### 3.4.1. Select Experienced Nurses to Save the Country From Disgrace

From the interviews, supporting the 76.4% of respondents who agreed that the selection should be done by the government, after engaging the participants on the “hows,” and the “whos” when it comes to the selection, it was found that the government should select nurses who have worked before because they have some experience. Thus, it was found that nurses who have worked before and have gained experience should be the ones selected to go. Some of the participants disclosed that selecting those with experiences will not disgrace the country when they are sent to work outside the country.

#### 3.4.2. Select Every Other Nurse

Some other participants disclosed that any nurse who has written the licensure exams and passed should qualify for the selection. Thus, nurses who have graduated and are unemployed should be included in the selection. The selection process should be opened for every qualified nurse to apply. The selection of nurses for export should not be limited to experienced nurses. This revelation confirms the practice in Kenya, where it is those unemployed nurses who are sent to work in the United Kingdom [26]. It was further revealed from the interview that the sending of nurses abroad and the selection should not be political. Thus, the process should not favour nurses with political networks, while those with any political link do not get the chance to also go. Also, it was revealed by some participants that the selection of the nurses should be done by an independent group to avoid political influences.

*Left to me alone, if any other government should say they need nurses from Ghana, the government of Ghana should select nurses who have worked before to go. My reason is that they have worked and have some experience, so when they go and represent the country, they won't disgrace the country. And as they are selected to go across the country, newly trained nurses like us are recruited into the system to practice, learn and gain more experiences (Kwame, a 24-year-old general nursing student from UCCSNM).*

*I think those nurses who have written their exams and passed and are not permanently employed should be selected to go. Or, the government can make it open for everyone to apply, and when some are selected to go, whether they were working or not, then replace those who were employed and gained the chance to go. Or the government can introduce some form of exam so that those who do well are selected (Akosua, a 25-year-old Midwifery nursing student from CCNMC).*

*The whole selection process should be fair. They should not make it political, where those who have political links will get the opportunity to go while the rest are denied. And concerning those to be selected, I think the whole process should be made open for anyone to apply, and based on some criteria that may be made available, the applicants are selected. It could be those who are working or those who are not employed. I think this will create an avenue to clear the unemployed nurses in the country (Mame Adwoa, a 24-year-old General nurse of CCNMC).*

#### 3.4.3. Nurses to Be Exported With Their Family

Furthermore, on the issue of selection and family inclusion, on the issue of allowing nurses to move with their family when going abroad to work, from Table, 405 respondents representing 83.9% agreed that the family of nurses (which in this study refers to the partners and children) should be included. The majority of the respondents agreed that nurses should be allowed to travel with their children as they go on government arrangements. This could be as a result of the fact that nurses would want to ensure that they have physical contact with their children and also give them the best treatment they desire for their children. Further, studies have revealed that in the case when children are left behind with the family while parents travel abroad, the children do not get the best care they deserve [27], and this could be why nurses would want to move with their families. Also, because children of migrant parents who are left in their home country face emotional problems that affect them psychologically [28] and also affect their education [29], nurses agreed that they will want to travel with their family. The interviews further revealed that some of the participants would love to go outside with their children when exported (Appendix, Qualitative instrument). They revealed that although that may bring some financial burden on the government, they will be happy if the government will work on the documentation for them to go and later bring their families with them.

*Personally, I will agree that the government will work out the documentation for nurses to send their partners or children, if possible, but they must not leave with the nurses at the very moment they will be traveling since that will be a burden for them at the initial stages of their work out there (Akosua, a 25-year-old Midwife of CCNMC).*

#### 3.4.4. Selection of Nurses Must Be Done by an Independent Group

On the contrary, 23.6% of the respondents did not support the idea of the government selecting the nurses, and 16.1% of the respondents did not support the inclusion of families of nurses in the programme. The interviews gave some insight as to why some respondents disagreed. From some of the participants, the selection of nurses to be exported should be done by an independent group or by a private body. The reason for this was to avoid the biases that come with a government body doing selections. It was revealed that when it is the government doing the selection, there will be some political imbalance that will enable some people getting selected and others being denied if it is the government doing the selection. Furthermore, some of the participants disclosed that they will not agree that the government should allow them to travel with their children and partners because it will cost the government.

#### 3.4.5. Financial Burden on Government to Include Family of Nurses

For these participants, sending nurses to go and work abroad with their husbands, wives and children comes with some financial burden on the government. In order not to burden the government with sending them along with their families, some of the participants suggested that the individual nurses could take that upon themselves if they would want to. The position of these participants shows the care they have for the government. Further, some of the participants disclosed that some immigration laws of some destination countries may not favour the decision to include families of nurses who are being exported. They also do not want to burden the government with too much cost. This finding could be explained by the SI theory that when the people perceive that the sending of their families will cost them, they will not want to take any action to do so.

*I don't think this one too will be become political. The selection must be done by an independent group that can be trusted. And the nurses who will be selected must be nurses who have either worked for two years or one year and have gained some experience (Mame Abena, a 24-year-old general nurse of UCCSNM).*

*Although someone will be happy about this, I will not be. I don't think this will be a good idea. The government of Ghana will be burdening the receiving country with all of these. I think that the arrangement should be done for only nurses. If in the future I will want to send any of my family members abroad, that will be my concern (Mame Adwoa, a 24-year-old General nurse of CCNMC).*

*I have a son, but if I am to travel on my own, I will not send him with me. And again, we must know that the country we are going to will have its laws on migration that may not allow us to come with our family. I will prefer to go and later come for them (Memuna, a 27-year-old enroled nurse of CCNMC).*

Concluding the discussion on the Table, there is a high appreciation on the side of the nurses for the government to put in place certain strategies to run the nurse exportation. They suggested that there should be some frameworks and policies to guide the export. It was also discussed that the institutionalisation of the export of nurses should have some defined norms and values to guide it. It came up from the findings that the government will have to arrange with other recruiting countries to send nurses to them. It was disclosed that operating the export of nurse exportation within bilateral agreement promises security. Also, it has been discussed in relation to the rotation of the nurses to be exported that rotating the nurses will help the government to plan and also serve as a check and balance. Finally, it has been discussed that the selection process should be either by the government or by some independent group. Although the findings of this study do not only support the export of nurses but also support smart strategies for the government to follow, the government of Ghana should prioritize staffing the local health sector with nurses before exporting surpluses.

## 4. Conclusion and Recommendation

Interestingly, the study has revealed nurses support for the export of nurses in Ghana although the government must have regard for policy and work within a framework, the government of Ghana will have to be strategic in exporting nurses so as the local health sector is not left orphan of nurses in the case of exporting for the money, and also leave the local health sectors malnourished, when nurses left behind are inexperienced. Notwithstanding, the details guiding every contract on the export of nurses should be made public as well as making public the data on nurses exported. It is important to clarify that the deployment of nurses to work internationally through bilateral agreements should not be characterised as modern-day slavery or the commodification of labour, despite some potential misconceptions. The current situation indicates that exporting nurses has become essential, serving as a practical approach for countries to create job opportunities for workers. Additionally, it offers financial benefits to expatriates and generates remittances for the government. This process cannot be classified as modern-day slavery or labour commodification, as the contracts signed by the government ensure the protection of workers' human rights and their right to work. The researcher recommends that the government stakes steps to engage stakeholders in the formation of a documented map for the export of nurses.

## Figures and Tables

**Table 1:** Government nurse export strategies.

Variables	Frequency	Percentage %
Policy and regulatory framework		

In order to export nurses, the government should institutionalise nurse migration.	Agree 432	89.4
	Disagree 51	10.6
It is important for the government to set up a regulatory body to facilitate nurse migration.	Agree 422	87.4
	Disagree 61	12.6
It will be in order for the government to develop a policy that will regulate nurse exportation.	Agree 419	86.7
	Disagree 64	13.3

Arranging with other countries and joining TiSA groups		

To send nurses abroad, the government should communicate with receiving countries.	Agree 414	85.7
	Disagree 69	14.3
The government must join groups like trade in service agreements to enable access to foreign markets.	Agree 414	85.7
	Disagree 69	14.3

Rotational issues		

The sending of nurses should be rotational to give other nurses the opportunity to offer their professional services abroad.	Agree 400	82.8
	Disagree 83	17.2
After 5 years, the government should bring back the nurses and send new sets of nurses.	Agree 384	79.5
	Disagree 99	20.5
The rotation should be done on a 5-year basis or more.	Agree 373	77.2
	Disagree 110	22.8

Selection issue and family inclusion		

The government should allow nurses to move with their partners and children, if any.	Agree 405	83.9
	Disagree 78	16.1
The selection process should be done by the government.	Agree 369	76.4
	Disagree 114	23.6

*Note:* Source: Field Data, 2023.

## Data Availability

The data that support the findings of this study are available on request from the corresponding author.

## Appendix A

1. Qualitative instrument.
  In-depth interview semistructured guide (for nurses).
  Hello, Good Morning/Good or afternoon. I am from the department of Sociology and Anthropology, University of Cape Coast. I am conducting a research on nurse exportation in Ghana. The aim of the research is to unearth the sociological undercurrent that surrounds the exportation of nurses. I would be grateful if you could make time out of your busy schedules to answer the questions below. You can be sure that any information you give will be kept private and used only for academic purposes. The discussion may last between 30 and 45 min. Thank you for agreeing to participate in this research.
  Demographics of Interviewee
i. Hello, can you tell me your name, age, level of education and children if any?
  Views on Opportunities in the Nursing Profession
1. Why did you choose to become a nurse?
2. Why do some nurses leave the country?
3. Have you heard of nurse exportation?
  Views on What Government Should
4. To control the nurses moving on their own, do you think the government should formalise migration of nurses?
5. So do you think the government should institutionalise nurses' exportation in Ghana.
6. If the government should institutionalise nurses' exportation, how should it be regulated?
7. Do you suggest that the government should expand the enrolment in the colleges if the government wants to send nurses outside to work?
8. Must the government arrange with other countries who need nurses in order to send nurses there?
9. Do you think that all nursing training colleges should be upgraded to degree level?
10. If the government is to send nurses abroad, do you think it should be rotational, and why?
  Views on the Selection Process
11. Who should be selected to go, and how should it be done?
12. How should the selection process be if the government should send nurses abroad?
13. Who should be selected to be sent abroad?
14. Which ministries or sectors should be involved in the exportation?
  Views on Benefits of Nurse Exportation to Nurses and Their Families
15. How beneficial will the sending of nurses be to nurses?
16. How does this also benefit your family?
  Views on Benefits of Nurse Exportation to the Government
16. Do you think the government stands to gain from exporting nurses abroad?
17. Will you agree to give some percentage of your salary to government if you are sent outside to work?
18. Will you agree to pay your tax to Ghana even when you are working outside?
19. What do you say about your salary paid into your Bank account in Ghana while you work outside?
20. Will you agree that government can use your money for investment and give them back to you later?
  NB: Is there any else you will want to share with me on the export of nurses?
